# Impact of Driver’s Age and Gender, Built Environment, and Road Conditions on Crash Severity: A Logit Modeling Approach

**DOI:** 10.3390/ijerph20032338

**Published:** 2023-01-28

**Authors:** Dongkwan Lee, Jean-Michel Guldmann, Burkhard von Rabenau

**Affiliations:** 1 Gangwon Institute, Chuncheon 24265, Republic of Korea; 2 Department of City and Regional Planning, The Ohio State University, Columbus, OH 43210, USA

**Keywords:** crash severity, at-fault drivers, age and gender, socio-economic factors, built environment, road conditions, logit model

## Abstract

The purpose of this research is (1) to investigate the relationship between crash severity and the age and gender of the at-fault driver, the socio-economic characteristics of the surrounding environment, and road conditions, and (2) to explain the probability of a bodily injury crash, including fatality, with the alternative being a property damage only crash. In contrast to earlier research that has focused on young and old drivers, age is considered here on its lifetime continuum. A logit model is adopted and the gender and age of the at-fault drivers are part of the independent explanatory variables. The unit of analysis is the individual crash. Since age is a continuous variable, this analysis shows more precisely how age impacts accident severity and identifies when age has little effect. According to the results, the type of vehicle, timing of the crash, type of road and intersection, road condition, regional and locational factors, and socio-economic characteristic have a significant impact on crashes. Regarding the effect of age, when an accident occurs the probability of bodily injury or fatality is 0.703 for female drivers, and 0.718 for male drivers at 15 years of age. These probabilities decline very slightly to 0.696 and 0.711, respectively, around 33 years of age, then very slightly increase to 0.697 and 0.712, respectively, around 47.5 years of age. The results show that age affects crash severity following a polynomial curve. While the overall pattern is one of a downward trend with age, this trend is weak until the senior years. The policy implications of the results are discussed.

## 1. Introduction

People tend to explain crashes with simple facts, such as a driver’s sex and age, and to relate crash severity to the built environment. However, the demographic characteristics of a driver in a car accident may have multiplier effects, for instance the fact that the driver is 20 years of age and male may lead to a higher-severity crash. Moreover, the surrounding environment near a crash site may have different effects on crashes. The environment represents primarily the built environment, infrastructure, or socio-economic characteristics of a neighborhood or community. For example, for older drivers with less ability to cope with high-density environments, complex situations may increase crash likelihood. On the other hand, young drivers with less experience and judgment, may produce greater crash incidence and severity in situations encountered more rarely, such as driving on a slippery iced road. Hence, it is necessary to investigate the interactions between a driver’s demographics and the surrounding environment in generating crashes of varying severity.

Regarding crash severity, crashes differ. Some are minor while others result in injury or death. Many factors impact the severity of a crash. This research identifies the factors that determine the severity of a crash once it has occurred, specifically the likelihood that it is a bodily injury or fatal (BI) crash, as opposed to a property damage only (PDO) crash. Unlike macro-level analyses that focus on the numbers of crashes in larger neighborhood units [1], micro-level analyses focus on an individual crash at a given location, such as an intersection. Recent research includes the creation of buffers around the crash point in order to investigate the direct surrounding environment.

It is not clear what variations in crash behavior would emerge if age appeared as a continuous variable in explaining crashes, or if age was analyzed for smaller age groups across the full age spectrum. As already noted, the disaggregate approach adds to the number of variables beyond those of the aggregate model, down to the precise vehicle type, driver condition including alcohol or drug use, and light, weather, and road conditions at the precise crash location. While the literature has analyzed how young and senior drivers differ from others, the classification of young and old drivers is somewhat arbitrary.

The literature, as summarized in Table 1, has looked at age as a factor in crash frequency, but has limited itself to the young (usually 24 years of age and younger) and the old (usually older than 65 or 70 years of age), and has contrasted their behavior with that of the remaining population. It is not clear that the particular age intervals chosen in the literature are appropriate, in the sense that behavior within the groups is homogenous and behavior between age groups is different. Moreover, in past research it is often not clear whether data pertain to all drivers involved in an accident or to drivers at fault in an accident. The latter is a subset of the former. The database available for this research provides information on drivers at fault. It is the characteristics of these drivers, the age, gender of those who cause the accident, that matter in explaining the accident.

The goal of this research is to better understand the interactions between a driver’s demographics and the surrounding environment in generating crashes of varying severity. For this, the age and sex of the guilty driver are taken as proxies of a driver’s characteristics. The focus is on the driver who is most responsible for a crash. Moreover, there are different built environments in urban and rural areas. Therefore, the overall environment for drivers and vehicles varies in these areas. The study area, in this research, provides enough variability in the built environment to uncover the influence of built-environment factors on crash severity.

The present approach is a micro analysis using logit modeling to estimate the likelihood that an accident is severe, i.e., results in injury or death. In a departure from earlier research, the focus is on the age and gender of the driver of the at-fault vehicle, and age is taken as a continuous independent variable, encompassing young, old, and middle-aged drivers. Other explanatory variables related to the crash, such as its location, vehicle attributes, road features, weather, and time, are also used as they are derived from the extensive crash reporting system. In addition, variables defined at the level of Traffic Analysis Zones (TAZs) are also considered to account for the impacts of the socio-economic characteristics of the neighborhood surrounding the crash location, including land uses. The remainder of the paper is organized as follows: Section 2 describes data sources and their processing and presents the modeling methodology. The results and their analysis are presented in Section 3. Section 4 includes discussions related to the results. Section 5 concludes and outlines areas for further research.

## 2. Materials and Methods

### 2.1. Data

**Figure 1:**
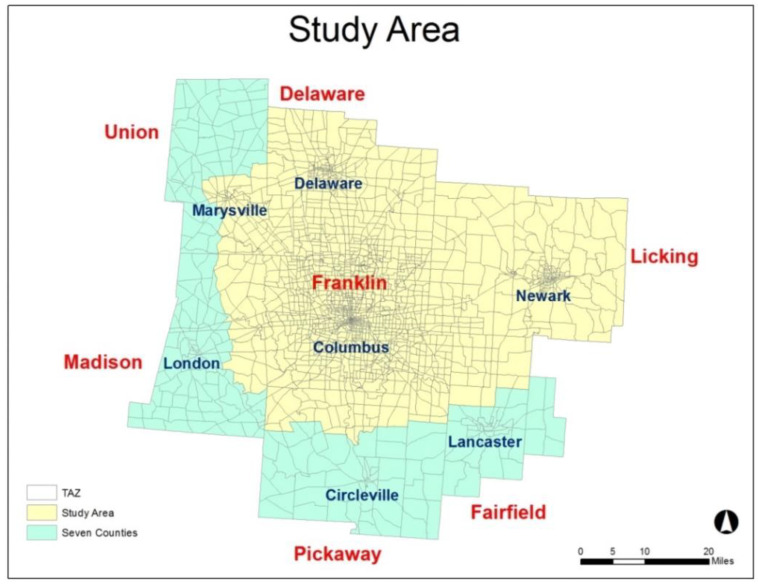
Central Ohio Region.

The data characterize the Central Ohio Region (part of the Columbus Metropolitan Area (CMA)), which includes Delaware, Fairfield, Franklin, Madison, Licking, Pickaway, and Union counties, and the City of Columbus, the capital of Ohio and the 15th largest U.S. city. According to the 2010 Census, its total population was 1,654,374. The Traffic Analysis Zone (TAZ) is selected as the geographical unit. The seven counties consist of 2052 TAZs. However, crash data are not available for some peripheral areas, hence only the area in yellow on Figure 1 is selected for this analysis. It is made up of 1805 TAZs and covers 87% of the area of the seven counties.

**Figure 2:**
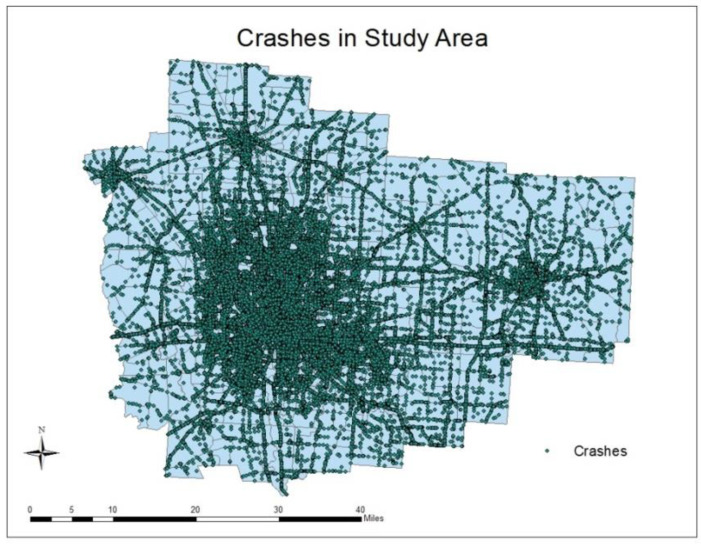
Individual crashes in the study area over 2006–2011.

About 284,611 crashes have occurred over 2006–2011 in the seven counties, 535,385 units (cars, motorcycles, etc.) were involved in these crashes, and 704,883 people were affected by them. The crash database was obtained from the Ohio Department of Public Safety (ODPS), and includes crash information, vehicle information, and people information. As the focus is on crashes where a vehicle and its drivers are at fault, such crashes are first extracted from the database, leading to the selection of 161,925 individual crashes. Figure 2 displays their spatial distribution. In addition to crash severity (fatalities, injuries, and property damages), a wide range of crash attributes are extracted from the ODPS database, such as at-fault driver’s sex and age, crash time, driving conditions, weather, and road conditions. The TAZ within which the crash took place is identified by overlaying the crash point locations with the TAZ map, using a Geographical Information System (GIS).

Socio-economic and built-environment data were obtained from several sources, are defined at the TAZ level, and are grouped into six categorizes as described below.

1. Regional and Locational Factors

These variables characterize the general crash location: urban versus rural, distance to the CMA center, and inclusion in the Mid-Ohio Regional Planning Commission (MORPC) transportation planning area. These data were obtained from MORPC. Infrastructure and surrounding driving conditions vary across these locations.

2. Socio-Economic Factors

These variables characterize population, employment, income, school enrollment, and housing. These data are all derived from MORPC and the 2010 Census of Population and Housing.

3. Land-Use Factors

Six land-use variables are considered: Agricultural, Commercial, Residential, Public Space, Industrial, and Undeveloped. These data were obtained from MORPC, which processed parcel-level data from County Auditors’ databases, are closely related to transportation flows generation and attraction.

4. Circulation and Network Factors

Public transit variables include numbers of bus stops and railroad crossings. Traffic flow variables include traffic volumes estimated with data obtained from ODOT, the Central Ohio Transit Authority (COTA), and the Delaware Area Transit Authority (DATA). Measures of car use, such as vehicle miles traveled per household, were derived from the National Household Travel Survey (NHTS).

5. Physical Environment Factors

These variables include number of bars, average age of buildings, area of historic districts, and size of shopping centers. These data were obtained from County Auditors’ databases and the National Register of Historic Places.

### 2.2. Modeling Methodology

Several types of models have been used in transportation choice research, such as the logit model [2,3,4,5,6], binary logit model [7,8] which uses a binary dependent variables (e.g., yes/no, pass/fail, win/ lose, etc.), ordered mixed logit model, which uses a discrete and ordinal dependent variable with more than two outcomes (e.g., customer satisfaction rating, etc.), mixed logit model [11,12,13,14,15,16,17], which assumes that the parameters vary from one individual to another, multinomial logit model [18,19,20,21,22,23,24,25,26], which uses a discrete and nominal dependent variable with more than two outcomes (e.g., red/blue/green, etc.), discrete choice model [48], which explain a choice from a set of two or more discrete alternatives. This research adopts the logit model to investigate how age impacts accident severity and to uncover when age has little effect, using age as a continuous variable.

The dependent variable Y is binary, set equal to 1 if the crash is BI-type, and to 0 if it is PDO-type. Fatal and injury crashes are regrouped as bodily injury (BI) crashes because of the small number of fatal crashes. The independent variables can be grouped into two groups of vectors: (1) variables specific to the individual crash, and (2) variables characterizing the TAZ where the crash took place.

The categories of crash-specific variables are:

- *X*_1_: age and gender of the at-fault driver;
- *X*_2_: vehicle type;
- *X*_3_: timing of crash;
- *X*_4_: crash location;
- *X*_5_: road condition;
- *X*_6_: other crash factors.

The categories of TAZ-related variables are:

- *Z*_1_: regional and locational;
- *Z*_2_: socio-economic;
- *Z*_3_: land use;
- *Z*_4_: circulation and network;
- *Z*_5_: physical characteristics.

The general form of the probability model is then:

P (*Y* = 1) = F (*X*_1_, *X*_2_, *X*_3_, *X*_4_, *X*_5_, *X*_6_, *Z*_1_, *Z*_2_, *Z*_3_, *Z*_4_, *Z*_5_) (1)

In order to assess the factors in the surrounding environment that can affect the probability of being injured or dead in a crash, a logit model is formulated:

(2) $$\mathrm{Pr}\left(Y=1\right)=\frac{exp(\alpha +\sum_{i=1}^{6}\beta_{i}+\sum_{j=1}^{5}\gamma_{j}Z_{j})\;}{1+exp(\alpha +\sum_{i=1}^{6}\beta_{i}X_{i}+\sum_{j=1}^{5}\gamma_{j}Z_{j})\;}$$

where,

α: constant;
$\beta_{i}$: coefficient for categories of crash-specific variables;
$\gamma_{j}$: coefficient for categories of TAZ-related variables;
$X_{i}$: categories of crash-specific variables;
$Z_{j}$: categories of TAZ-related variables.

## 3. Results

### 3.1. Descripctive Statistics

Descriptive statistics for the crash-specific variables used in the logit model are presented in Table 2. Descriptive statistics for the TAZ-level selected variables are presented in Table 3.

### 3.2. Results of Logit Model

The relationship between each potential explanatory variable and the dependent variable was first investigated with one-variable logit models. Variables with at least a 10% significance level were retained. Next, these variables were combined into a unique logit model, and only those variables significant at the 10% level were retained. The estimated parameters for the selected logit model are presented in Table 4.

The following analyses focus on the model in Table 4, which includes 43 explanatory variables, all except four significant at the 0.01% level.

### 3.3. Age and Gender of the Culpable Drivers

Both the AG and MD variables were initially significant, with a negative and positive sign, respectively. As the AG variable is continuous, higher-order terms were introduced, and both the square (AG^2^) and cube (AG^3^) of AG also turned out to be very significant. However, cross-products between these variables and MD turned out insignificant, and were discarded. In order to better assess the effect of AG, the polynomial function F made of the three age-related variables in Table 4 is analyzed, with:

F= −0.0134AG + 0.000344AG^2^ − 0.00000285AG^3^ (3)

In order to assess the variations in F, its first-order derivative is computed:

(4) $$\frac{dF}{dx}=-0.0134+0.000688\mathrm{AG}-0.00000855{\mathrm{AG}}^{2}$$

The derivative has a value of zero at the following two values of AG:

AG_low_ = 33.06

AG_high_ = 47.41.

**Figure 3:**
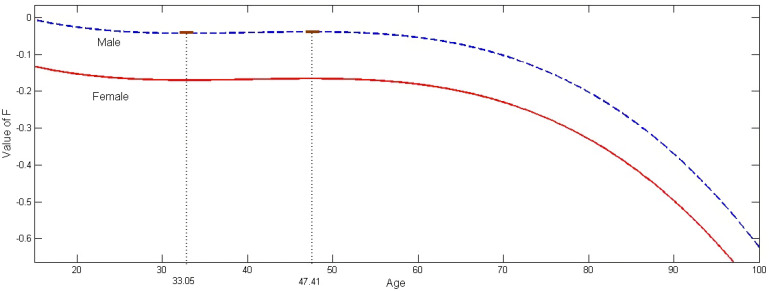
Age and the value of F.

The value AG_low_ corresponds to a minimum, and the value AG_high_ to a maximum in the F curve, as illustrated in Figure 3. Equation (3) represents the “Female” curve. The “Male” curve is obtained by adding the coefficient 0.1269 to Equation (3).

For both male and female drivers, F declines slightly from AG = 15 to AG = 33.06, then increases very slightly to AG = 47.41. From that point on, F declines precipitously. However, while the variations in F are a reflection of the variations in the probability of a crash being BI-type, they are not the same, and it is necessary to compute these probabilities for the same AG range. To do so, it is necessary to select values for all the other variables appearing in Table 3. For the sake of simplicity, all the other independent variables are set at their mean values. The simplified probability functions are then:

(1). Male Drivers

(5) $$\mathrm{Pr}\left(Y=1|AG\right)=\frac{exp\left(-0.0134*AG+0.000344*AG^{2}-0.00000285*AG^{3}+1.0697\right)}{1+exp\left(-0.0134*AG+0.000344*AG^{2}-0.00000285*AG^{3}+1.0697\right)}$$

(2). Female Drivers

(6) $$\mathrm{Pr}\left(Y=1|AG\right)=\frac{exp\left(-0.0134*AG+0.000344*AG^{2}-0.00000285*AG^{3}+0.9968\right)}{1+exp\left(-0.0134*AG+0.000344*AG^{2}-0.00000285*AG^{3}+0.9968\right)}$$

**Figure 4:**
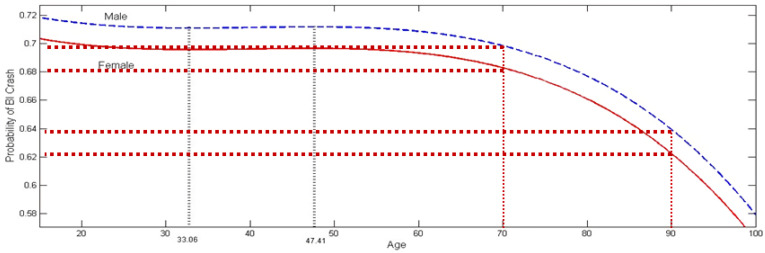
Age and probability of BI crash.

The corresponding probability curves are presented in Figure 4.

At age 15, the probability of the crash BI is 0.703 for female drivers, and 0.718 for male drivers. These probabilities decline very slightly to 0.696 and 0.711, respectively, around age 33.06, then very slightly increase to 0.697 and 0.712, respectively, around age 47.41. From that point on, the two curves first decline very slightly up to age 70, after which the decline accelerates. At age 80, the female and male probabilities are 0.661 and 0.677, respectively, and at age 90 they are equal to 0.622 and 0.639, respectively.

The above results are somewhat surprising, as one would have expected more differentiated probabilities. While the BI crash probability is highest for the very young drivers (15–20), it is not very different from the probabilities for adulthood years. As expected, the probability declines significantly for senior drivers, but mostly in late years. In summary, except for these very senior drivers, age does not appear to strongly affect the probability for a crash to be of the BI-type.

The crash literature has heavily focused on driver age as influencing crashes, and in particular how age affects the likelihood of crashes among teenage and senior drivers. However, the present results differ in at least two ways: The analysis looks at all age groups in a continuous manner, and hence can identify the age at which crash incidence changes. In the literature, the cut-off points for the young- and old-age cohorts are assumed rather than being the result of an analysis. Second, the literature does not explicitly deal with drivers at fault, and hence it is difficult to identify the extent to which a particular age group is involved in accidents or causes accidents as faulty party. In a general way, the present results confirm some of the findings in the literature. They suggest that over the middle age range, crash probabilities do not change much and hence the literature is correct in focusing on the young and the old, for which severe crash probability differs. Further, the literature suggests that male drivers are at a greater risk of BI accidents and women are more involved in non-fatal crashes [49,50]. This is confirmed by this study.

### 3.4. Type of Vehicle

The benchmark is a mix of trucks, taxi, emergency, motorhome, equipment, and other vehicles, as well as trains. Accidents in which at-fault drivers drive compact, mid-size, or full-size vehicles are less likely to result in BI than those involving benchmark vehicles. Similar results hold for vans, SUVs, pickup trucks, and motorcycles. However, bus-type vehicles have a positive sign, which indicates that they are more likely than the benchmark vehicles to generate BI crashes. The effect of vehicle type on accident severity has not yet been much studied in the literature. An exception is [42], which considers the special case of pedestrian crashes and finds that emergency vehicles and motorcycles/mopeds have a positive impact, and trucks, vans, and buses a negative impact on such crashes. These results are somewhat counterintuitive. The present results have much greater generality as they do not focus only on pedestrian crashes.

The result for motorcycles is interesting. The coefficient (−2.97) is much larger (in absolute term) than those for all the other vehicle-type variables. Usually, riding a motorcycle is considered more dangerous than riding a car. Motorcyclists are not protected by the body shell of a car or other safety attributes, such as seatbelts or airbags. The driver’s body is directly exposed to the impact of a car or obstacle. The results suggest that if the motorcyclist is the culpable driver in a crash, body injuries are less likely than if the driver of a car were the at-fault driver. While this seems counterintuitive, there are possible explanations. First, BI crashes are not limited to accidents in which the at-fault driver suffers injuries. Much of the time it is others, passengers in the vehicle of the at-fault driver, pedestrians, or passengers in other vehicles, that suffer injury. Comparing the vulnerability of at-fault drivers is therefore besides the point. It is the individual vulnerability and the number of all participants in an accident that matters. Hence the result can be explained, all else being equal, if the number of participants in a crash caused by a motorcycle were smaller than the number caused by a car. The same is true, again all else being equal, if the vulnerability of participants other than the crash-causing driver is smaller in a crash caused by a motorcycle than by a car. The number of crash participants is likely to vary between the two types of accidents. Given the greater instability of 2-wheel vehicles over 4-wheel vehicles, motorcycles are more prone to accidents that involve only the motorcycle. These types of accidents would automatically fault the motorcyclist. However, quite a number of such accidents may not result in injury, as the driver anticipates the accident.

### 3.5. Timing of Crash

Seasonal variables are associated with the type of crash. If a crash occurs in summer, the odds of a BI crash are smaller than in spring or autumn (benchmark seasons). On the other hand, winter increases the odds of a BI crash. In summer, driving conditions may be better than in other seasons in the Central Ohio Region due to the summer break in schools and colleges. The summer break lessens traffic congestion and may calm traffic. Driving during the winter season increases the probability of a BI crash. Ice, slippery roads, and low temperatures may affect a driver’s physical conditions, resulting in worse vehicle maneuverability.

The results also show that driving on Friday may lead to more BI crashes. Friday is the end of the week, which may make people more relaxed, and drivers less attentive. People may participate in parties or other nightly events, which may also contribute to more BI crashes.

With respect to time of day, driving during morning peak hour (7:00–9:00 am), night peak hour (5:00–7:00 pm) and from 12:00 pm to 5:00 pm increases the likelihood of a crash to be BI-type. The morning and evening rush hours, and to some extent the lesser peak hours of the lunch break, are characterized by congestion. Drivers frequently change lanes, do not keep a minimum distance to other cars, and encounter difficult situations when entering and exiting highways or turning on multi-lane arterial roads. The literature indicates that these conditions raise accident rates, and it is not surprising that they raise the rate of BI crashes. Morning and evening rush hours also take place at least in part during twilight and dawn hours when visibility is reduced. See [51] for results on this effect.

### 3.6. Type of Road and Intersection

The benchmark cases include 5-point intersections, crossovers, railways, shared paths, not an intersection, and others. Compared with the benchmarks, 4-way intersections and driveways are less likely to lead to BI crashes, whereas roundabouts and ramps are more likely to do so.

At 4-way intersections, drivers generally must stop their car or reduce speed before turning or proceeding ahead, and this caution leads to less BI crashes. The higher likelihood of BI crashes at roundabouts may be due to less visibility of other cars entering the roundabout. The results imply that when a crash occurs, it is more likely to be BI-type than at other intersections. However, overall, the number of crashes may be much lower and hence the incidence of BI crashes at roundabouts is lower than at other intersections. The results indicate that ramps increase the likelihood that a crash is BI-type. Vehicles at high speed on the highway need to reduce their speed abruptly or need to change lane suddenly to enter the exit ramp. A ramp with a sharp curve and a 25 m/h speed limit or steep slope, may make it difficult to control the vehicle. Using a ramp to enter a highway may also be dangerous, particularly if high-speed vehicles on the highway do not provide some leeway for the entering cars. Crashes that occur at driveways are more likely to be PDO crashes due to the low speed of the vehicle entering or exiting the driveway.

### 3.7. Road Conditions

Road shape can be an influential factor regarding the type of crash. When the road contour is curved, whether level or grade, a BI crash is less likely, possibly because of the need to reduce the driving speed. The results also show that road conditions may be influential factors. A road covered by ice increases the odds of a BI crash. The benchmark includes all other conditions (dry, wet, snow, etc.). This result is likely related to the difficult maneuverability of cars under icy road conditions. Moreover, ice conditions may overlap with other conditions, such as rain or snow, which can reduce the visual field. Drivers often inadequately compensate for such difficult conditions, perhaps because these conditions are not always recognizable, because compensation is not feasible, or because of a lack of experience with these conditions. Snow in southern states will likely result in much greater accident severity than snow in Canada. Several studies confirm the positive effect of bad weather on crashes, for instance [42] for vehicle–pedestrian crashes and [52] for crashes resulting in injury or fatalities.

In contrast to expectations, poor light and visibility conditions do not necessarily contribute to crash severity. Darkness decreases the odds of bodily injury crashes. Apparently, this is yet another situation when drivers may compensate by driving more cautiously or at a reduced speed, limiting accident severity even if accident likelihood rises. The authors of [51] suggest that the smaller the daylight minutes during rush hours, the greater the number of crashes.

### 3.8. Alcohol, Drug, and Work Zone

The results indicate that alcohol- and drug-related crashes are more likely to be PDO than BI crashes. This is quite an unexpected result, but there might be a possible explanation: when drivers are influenced by alcohol or drugs, there are more chances that a crash will happen, but since they are mentally and physically exhausted under the influence, they may drive more slowly and in zigzag, hitting objects and properties. Moreover, surprisingly, pedestrian-related crashes are more PDO than BI-type. However, in such pedestrian-related crashes, the pedestrian is not the at-fault party, and it may be that the driver, trying to avoid a pedestrian, may swerve suddenly and hit property rather than the pedestrian.

Finally, accidents in work zone areas are more likely to result in BI. Workers operate under dangerous conditions, where they can be struck by cars. Inexperienced or incautious drivers may run over the work zone. Driving lanes are often narrow, and may change in unexpected ways, which makes it difficult for drivers to fully adjust their driving. There is also asymmetry of traffic participants, as workers must work in the immediate vicinity of traffic, yet lack protection.

### 3.9. Regional and Locational Factors

An increasing distance from downtown Columbus increases the likelihood of a crash being BI. The closer to downtown, the greater the level of congestion and the slower the traffic. While accidents may be more frequent closer to downtown, the likelihood that they result in injury declines. This holds despite the greater asymmetry in street participants, and in particular the greater presence of vulnerable pedestrians. More complex signage, more pedestrians, and more law enforcement officers may lead to more careful driving. The urban dummy variable has a positive and significant coefficient. In contrast to expectations, an urban environment raises the odds of a BI crash. In urban areas, there are many attractions which can capture drivers’ attention in contrast to rural areas, and this may lead to more serious crashes.

### 3.10. Socio-Economic Factors

Population density, retail service employment, household income, and the proportion of Whites, all increase the likelihood for a crash to be BI-type. Denser areas with a lot of commercial activities may be more difficult to drive in, increasing the likelihood of BI crashes. It is not clear why a large share of Whites and higher household income increase the likelihood of BI crashes. Unexpectedly, when a neighborhood has a high proportion of young (Age < 14) or older (Age > 50) residents, the likelihood that an accident is of BI-type declines. Both younger and older residents are more vulnerable than middle-age adults, particularly as pedestrians. The young are less familiar with traffic dangers, older residents react more slowly, and both young and old often act impulsively and without proper precaution, so the result is unexpected. There are a number of possible reasons, though all remain speculative without additional data. As elsewhere, drivers in potentially vulnerable neighborhoods may overcompensate, resulting if not in fewer accidents, at least in accidents that are less often BI-type. Neighborhoods with preponderance of young or senior populations may differ from others in ways not captured by other physical neighborhood characteristics, making them safer in terms of BI. Future research will have to identify these reasons.

### 3.11. Land-Use

The results show that crashes in predominantly agricultural and residential areas are less likely to be BI-type. There may be less traffic in agricultural areas, hence less opportunities for severe accidents. Residential areas require lower speeds and more stops, which reduces the likelihood of BI crashes.

### 3.12. Neighborhood Physical Factors

The results show that physical neighborhood characteristics are associated with the possibility of a crash being the BI-type. The average age of buildings has a negative sign. Older neighborhoods may require more careful driving, leading to less BI crashes. However, the Historic District variable has the opposite effect, for which there is no clear explanation. The size of shopping centers has a positive effect on the odds that an accident is BI-type. This can be attributed to the difficulty of driving in such areas.

## 4. Discussion

This research tried to identify the variables that determine whether a crash results in Bodily Injury (BI) or Property Damage Only (PDO). BI crashes are of interest because they are generally more serious and costly than PDO crashes. The analysis focused on driver attributes, physical neighborhood characteristics, neighborhood resident and employment populations, and other factors such as road and weather condition. It adds significant detail to driver and vehicle attributes, and crash-site and weather conditions. One finding is that age affects crash severity following a polynomial curve. While the overall pattern is one of a downward trend with age, this trend is weak until the senior years. Among vehicle types, bus-type vehicles significantly increase the probability of a crash being BI, while motorcycles have the opposite negative effect. The results indicate that seasonal, weekly, and daytime variables are associated with the probability of BI crashes. Crashes that occur in summer are less likely to be BI crashes, while those in winter are more likely to be so. In terms of day of the week, driving Friday contributes to a crash being BI-type. Regarding the time of day, driving during morning and night rush hour increases the probability of a crash being BI-type.

The type of road and road conditions are also important factors for crash severity. Crashes that occur at 4-way intersections and driveways are less likely to be BI-type. Ramps and roundabouts have the opposite effect. Driving on icy roads is more likely to result in crashes being BI-type. Unexpectedly, driving under poor light conditions has the opposite effect. Among other factors influencing the likelihood of a crash being BI-type, alcohol and drugs have a negative effect, whereas work zones have a positive one.

Regional and locational factors, such as the distance to the center of the city of Columbus and the urban character of a TAZ positively and significantly impact the likelihood of a crash being BI-type. Neighborhood socio-economic conditions also matter. The higher the proportion of residents under 14 and over 50, the lower the likelihood of a crash being BI-type. Higher population density and larger retail service employment have the opposite effect. Two land uses, agricultural and residential, are negatively associated with BI crashes. Finally, the likelihood of a BI crash increases at lower posted limits, particularly less than 45 mph.

The research has shown that TAZ socio-economic characteristic have a significant impact on crashes. Yet not much is known about the mechanisms by which they affect crashes. Three such mechanisms are suggested, the effects that TAZ socio-economic conditions have on (i) the local composition of drivers at fault, (ii) the density and type of street activities in its area, and (iii) the built environment. However, some of the variables, such as the share of younger or older residents or the share of the White population, can influence accidents through all three mechanisms, and it is not clear which is most important. Indeed, we do not know the extent to which TAZ residents are also TAZ drivers at fault, or the extent to which TAZ residents are involved in crashes in their own neighborhood. Additional data may permit to shed light on these questions.

## 5. Conclusions

The modeling approach explained crash severity by combining explanatory variables related to the neighborhood (TAZ) of the crash with variables related to individual driver behavior, vehicle characteristics, and crash environment, such as weather, time of day, visibility, and road curvature. One contribution of this approach was to identify the age effect on crash severity. In approximate terms, the likelihood that a crash is severe does not change much for at-fault drivers between the age of 25 and 55, suggesting that limiting the study of age effects to the young and old, plus a single group in between may be sufficient for most purposes. The model is used to explain the probability of a bodily injury crash, including fatality, with the alternative being a property damage only crash. Because the age of the at-fault driver is taken as a continuous independent variable, it is possible to assess the lifetime impact of age, for both male and female drivers. According to the results, when an accident occurs, the probability of a bodily injury or fatal crash is 0.703 for female drivers, and 0.718 for male drivers at 15 years of age. These probabilities decline very slightly to 0.696 and 0.711, respectively, around 33 years of age, then very slightly increase to 0.697 and 0.712, respectively, around age 47.5 years of age. The results also confirm suggestions in the literature that males are more aggressive drivers and take greater risks. Male drivers of all ages are more likely to produce a crash leading to injury or death than female drivers.

Many variables that impact crashes and crash severity are beyond the control of planners or transport engineers. Even these variables may generate policy implications. For example, crashes are more likely in urban than rural areas, and crashes diminish with distance from the city center. While beyond control, they suggest transporting planners that, given limited budgets, it is wise to invest scarce safety resources in urban areas and close to the city center rather than in more distant rural areas. They also suggest the need to better understand just exactly what lies behind the differences in crash incidence. The most obvious reason is the higher traffic volume and the greater population and employment densities in urban than rural areas. As these variables were controlled for, other urban attributes must be the reason and will have to be investigated.

The research conducted in this study suggests numerous avenues for follow-up. The database developed for this research has the potential for a wide range of follow-up studies. One aim of the research was to introduce the at-fault driver into the analysis of crashes, as opposed to other crash-involved drivers. While the research succeeded in this aim, future research should analyze how the two types of analysis differ. The database allows for an analysis of crash-involved drivers, crash at-fault drivers, and the residual drivers that are involved but not at-fault. Fault is rarely quite clear-cut. Rear-end crashes, for example, are always taken to be the fault of the rear-ending vehicle, though the behavior of the front vehicle often contributes to this type of crash. It would be of interest to see whether there is a significant difference between these sets of drivers, and how they differ. This would also help in interpreting existing studies that have mostly focused on crash-involved drivers.

This research has some limitations that need to be addressed in future research. First, the data cover the period 2006–2011. Changes in vehicle safety standards, improvements in road infrastructure (e.g., roundabouts), changes in local enforcement, and expanded driver and pedestrian education may have since modified the roles of the various factors considered in this study. In particular, the COVID-19 pandemic may have influenced the overall environment for drivers, pedestrians, and traffic conditions. In this change context, it is noticeable that road fatalities in the U.S. have increased from 30,000 in 2011 to 36,000 in 2020 “https://www-fars.nhtsa.dot.gov/Main/index.aspx (accessed on 24 January 2023)”. Second, more spatial attributes need to be considered by adopting more advanced spatial analysis techniques.

## Figures and Tables

**Table 1:** Contents of the literature.

Models	Variables	Main Results	Reference
Logistic model	Accident severity, namely, location, cause of accident, etc.	Contributing factors to accident severity, with data derived from a sample of 560 crashes in Riyadh, Saudi Arabia. Location (intersection) is closely associated with crash severity.	[2]
	Hit-and-run crashes, driver characteristics, vehicle types, crash characteristics, roadway features and environmental characteristics.	Identifies the associated factors of hit-and-run crashes in Singapore. Male drivers, drivers aged between 45 and 69 years, two-wheel vehicles are more associated with these crashes.	[3]
	Road design elements (vertical and horizontal curves) road access (bus stops, public and private access lanes) and land use (gas stations, parking places).	Contributing road characteristics to crashes severity in Poland. The type of shoulders on both sides of a roadway, area type, pedestrian sidewalks, and intersection significantly influence crash severity.	[4]
	Driver characteristics, road condition, collision type, safety equipment usage, driver ejection, alcohol involvement, speed limit.	Examines the relationship between crash severity and the characteristics of gravel roads, using data over 1996–2005 in Kansas. Safety equipment usage, alcohol involvement, speed limit, and driver-related factors, have significant influences on crash severity.	[5]
	Educational attainment, median housing value, gender and age, median housing value, rurality percentage at the zip code level.	Investigates whether the socioeconomic characteristics of a driver-based residence zip code have any relationship with the likelihood of post-crash medical services.	[6]
Binary logit model	Wind data, overturning truck crash.	Wind speed is a critical factor in overturning freight vehicle crashes in Wyoming.	[7]
	Road (location, street, paving stretch, surface, signposting), external environment (day of week, weather, hour, season), driver (age, gender, license), accident (crash type, vehicle, dead).	It is found that male drivers are more likely to be involved in fatal crashes in an intersection crash than female drivers. Drivers aged 65 years and above are more likely to be involved in fatal intersection crashes than other age groups. Drivers aged below 45 years have lower probability to be involved in a front/side collision.	[8]
Generalized logit model	Railway features, highway features, crossing features, traffic controls, and land use.	Key factors for crash severity at a railroad grade crossing using logit analysis. The number of daily trains and the presence of a law enforcement camera can influence crash severity.	[9]
Ordered mixed logit model	Driver, traffic, crash-related and vehicle characteristics.	Certain factors including tripped rollovers can increase chances of fatal injury and injuries can be sustained by moped riders.	[10]
Mixed logit model	Roadway characteristics, vehicle attributes, and driver behavior.	The effect of the use of safety belts in single and multi-occupant vehicles in Indiana. Safety belt is associated with vehicle type, gender, time, but these effects vary across the population.	[11]
	Average daily traffic per lane, average daily truck traffic, truck percentage, interchanges per mile and weather effects such as snowfall, the number of horizontal curves, number of grade breaks per mile and pavement friction.	Volume-related variables are good for random parameters, while roadway-related parameters are good fixed parameters for these crashes.	[12]
	Roadway characteristics, vehicle attributes, and driver behavior.	The possible unobserved heterogeneity in pedestrian injury severity caused by motor vehicle crashes in North Carolina. Darkness, truck, freeway, and age of pedestrians can increase the possibility of fatal injury in pedestrian-related crashes.	[13]
	Roadway-surface conditions, driver’s age, gender.	Drivers in different age and gender groups perceive and react to road-surface conditions in different ways, which may result in varying crash severity.	[14]
	Driver characteristics (age, gender.), vehicle attributes, and roadway characteristics (light condition,).	Driver injury severity in single-vehicle crashes in California, focusing on the heterogeneous effects of age and gender. Male driver, drunk driving, unsafe speed, older driver, driving older vehicle and driving in darkness without streetlights can increase the probability of fatal crashes.	[15]
	Traffic volume, distance of the crash to the nearest ramp, and detailed driver’s age, vehicle types, and sides of impact, etc.	At-fault driver’s influential factors on crash severity on urban freeways in Florida. Age, traffic volume, distance of the crash to the nearest ramp, vehicle type, side of impact, and percentage of trucks are important.	[16]
	Manner of collision, motorcycle rider and non-motorcycle driver and vehicle actions, roadway and environmental conditions, location and time, motorcycle rider and non-motorcycle driver and vehicle attributes, etc.	Adopts mixed logit models to investigate the effects of crash factors on crash severity. Non-uniform effects of rear-end collisions, roadway speed limit, type of area, riding season, motorcyclist’s gender, light conditions, roadway surface conditions, helmet use influence on crash severity.	[17]
Multinomial logit model	Environmental factors, roadway conditions, vehicle characteristics, and rider attributes.	Investigates the influential factors on crash severity (five levels) in single-vehicle motorcycle accidents, addressing the need of multinomial logit formulation.	[18]
	Driver characteristics (age, gender.), single-vehicle accidents involving passenger cars.	The effect of driver age and gender on crash injury severity in single-vehicle crashes. There are significant different behavioral issues between genders in different age groups	[19]
	Roadway characteristics, accident types, weather conditions, etc.	Weather condition is frequently associated with roadway safety.	[20]
	Road characteristics, vehicle attributes, and driver behavior.	Crash injury severity and influential factors (crosswalk spacing, presence of both horizontal and vertical curves), vehicle type, signal timings, alcohol, gender, light condition, weekend.	[21,22,23,24]
	Pedestrian age, male driver, intoxicated driver, traffic sign, commercial area, darkness with or without streetlights, sport-utility vehicle, truck, freeway, two-way divided roadway, speeding-involved, off roadway, motorist turning or backing, both driver and pedestrian at fault, and pedestrian only at fault, etc.	Develops heteroskedastic logit analysis to investigate the influential factors associated with the injury severity of pedestrians in motor-vehicle crashes in North Carolina. Pedestrian age can increase the probability of fatal injury and it grows more pronounced with increasing age past 65 years.	[25]
	Monetary cost factors, time cost factors, throughputs, airports, etc.	In terms of the methodology, it adopts a multinomial logit model. It shows that reducing air cargo connecting time at an airport via adequate investment in capacity is important in terms of saving time costs.	[26]
Standard multinomial logit and mixed logit models	Roadway characteristics (intersection and non-intersection), vehicle attributes, and driver characteristics (bicyclist, wearing a helmet, drugs, alcohol).	Finds that (1) driving under the influence of drugs or alcohol, (2) striking the side of the bicycle, and (3) crashes involved with a heavy-duty truck can increase the likelihood of severe injuries from motor vehicle crashes at intersections and non-intersection.	[27]
Multinomial logit model and latent class logit model	Single-vehicle crashes on rural roads.	Uses multinomial logit model and latent class model at the same time, finding that vehicle age and surface condition (such as dry, wet, or icy) do not significantly impact driver injury severity.	[28]
Ordered probit model	Road characteristics, vehicle attributes, driver behavior, and driver characteristics.	Uses ordered probit models for crash injury severity analysis to examine the factors that affect the risk of different injury levels sustained under various types of crashes including two-vehicle crashes, single-vehicle crashes, motorcycle, pedestrian, etc.	[29,30,31,32,33,34]
Bayesian ordered probit models	Driver’s characteristics, vehicle type, and roadway conditions, etc.	Introduces Bayesian ordered probit models and compares the results with those of ordered probit models. When the sample data size is small, the Bayesian ordered probit model can produce better prediction performance than the ordered probit model.	[35]
Multinomial probit model	Gender of the motorcyclist, speeding, use of alcohol and/or drugs, helmet use, being involved in a single-vehicle crash or at a non-intersection location, horizontal curves, graded segments, major roadway.	Finds that being a female motorcyclist, excessive speeding, use of alcohol and/or drugs, and riding without a helmet significantly increases fatality and severe injury in terms of in a single-vehicle crash, at a non-intersection location, on horizontal curves or graded segments, and major roadways.	[36]
Multinomial logit, ordered probit, and mixed logit	Environmental factors, vehicle attributes, and driver characteristics.	Uses three commonly used methods, multinomial logit, ordered probit, and mixed logit, to investigate the effects of under-reported crash data. Fatal crashes must be the baseline severity for the MNL and ML models to minimize the bias and the variability of a model. The rank for the crash severity must be from fatal to property damage only in a descending order for the ordered probit models.	[37]
Ordered probit model	Impact of vehicle, occupant, driver, and environmental characteristics.	Investigates the influential factors of large truck crash severity using ordered probit models. Finds that the likelihood of fatalities and severe injury rises with the number of trailers, while it falls with the truck length and gross vehicle weight rating.	[38]
Ordered probit, ordered logit, and multinomial logit model	Time, emergency service arrival time, crash location, primary crash factors, weather, radius of curvature, vertical grade, type of vehicle at fault, driver age.	The time between midnight and 6:00 a.m.; driving while drowsy; median violation; car versus car collision; car versus people collision; car only collision; two or more related vehicles involved; van are the factors increasing risk of severe accident. Time between 6:00 A.M. and noon; ramp; toll gate; vehicle defects; obstacles and poor road conditions; rainy or snowy weather are the factors decreasing risk.	[39]
Spatial analysis and negative binomial regression	Roadway characteristics and spatial/land use, vehicle attributes, and driver characteristics.	Incomplete sidewalks and high crosswalk density are associated with pedestrian crash risk. People perceive a lower risk near university libraries, stadiums, and academic buildings.	[40]
Multivariate models	Spatial/land use, transit access, commercial access, and population density, built environment and design characteristics.	Examines both risk exposure and injuries sustained in child pedestrian-vehicular crashes in the vicinity of public schools. There is a significant association with several built-environment and design characteristics.	[41]
Generalized ordered probit model	Environmental characteristics on the severity of injuries sustained in pedestrian–vehicle crashes	It is found that women pedestrians tend to be injured less frequently than male pedestrians; children have an increased likelihood of injuries; older persons are more likely to be fatally injured in pedestrian–vehicle crashes.	[42]
Generalized linear model and negative binomial model	Pedestrian crashes and demographic (population and household units) and socio-economic characteristics (mean income and total employment), land use), and accessibility to public transit systems, road network characteristics (the number of lanes, speed limit, presence of median, and pedestrian and vehicular volume).	Population, transit stops, and pedestrians can increase pedestrian crashes. On the other hand, single family, urban residential commercial and neighborhood service can lower pedestrian crashes. Demographic, socio-economic, land use, and road data are better predictors than traffic data. Buffers of 0.5 mile yield better estimates for all and low activity intersections, while 1 mile buffers yield better estimates for high activity intersections.	[43]
Visual inspection of Google Street View and logit model	Presence of sidewalks, buffers between the road and the sidewalk, street lighting, number of travel lanes and the presence of medians, traffic controls at intersections, and posted speed limits.	Lack of sidewalks, buffers, high-speed roads, roads with six or more lanes, lack of traffic lighting, speed are associated with severity of pedestrian casualties. Age of pedestrians can cause more severe casualties.	[44]
Machine learning techniques	Survey data	In terms of Work-related Musculoskeletal Disorders (WMSDs), it is found that several risk factors (involvement in physical activities, frequent posture change, exposure to vibration, egress/ingress, duty breaks, and seat adaptability issues) influence the frequency of pain of drivers.	[45]
Multinomial logit model and discrete choice model	Mode choice preference data collected from airport passengers (540 observations).	Discrete choice model optimization algorithms using Excel is proved to be efficient in managing model tasks. Maximum likelihood method is an optimal method for estimating the coefficients of the variables. Newton Raphson is one of the best algorithms, while the worst performed algorithm is the Steepest Ascent (SA) method.	[46]
Review		Presents a complete review regarding analytical methodologies of crash injury severity models and approaches in highway accident research.	[47]

**Table 2:** Descriptive statistics of the selected individual crash variables.

Category	Variable	Description	Mean	Std Dev	Minimum	Maximum
Crash Severity	Bodily Damage	If the crash resulted in injury damage or fatality then = 1, else = 0	0.296	0.457	0	1
Sex	MD	If driver is male = 1, if not = 0	0.574	0.494	0	1
Age	Age	Driver’s age	36	16	16	105
	Age^2^	Driver’s age^2^	1575	1460	256	11,025
	Age^3^	Driver’s age^3^	80,391	112,918	4096	115,7625
Type of Vehicle	Compact	If type of unit is sub-compact, compact then = 1, else = 0	0.172	0.377	0	1
	MidSize	If type of unit is midsize then = 1, else = 0	0.329	0.47	0	1
	FullSize	If type of unit is full size then = 1, else = 0	0.101	0.301	0	1
	Van	If type of unit is mini-van or van then = 1, else = 0	0.081	0.273	0	1
	SUV	If type of unit is SUV then = 1, else = 0	0.156	0.363	0	1
	Pickup	If type of unit is pickup then = 1, else = 0	0.1	0.3	0	1
	Truck	If type of unit is single unit truck, trailer, truck tractor, truck trailer with double short or long, converter dolly, semi-trailer, fifth wheel, tractor with triples then = 1, else = 0	0.042	0.201	0	1
	Motorcycle	If type of unit is motorcycle then = 1, else = 0	0.007	0.083	0	1
	Bus	If type of unit is school bus, church bus, public bus, or other bus then = 1, else = 0	0.006	0.079	0	1
Time	Afternoon	If time is between 12 pm and 5 pm then = 1, else = 0	0.362	0.481	0	1
	Peak Time1	If time is between 7 am and 9 am then = 1, else = 0	0.111	0.315	0	1
	Peak Time2	If time is between 5 pm and 7 pm then = 1, else = 0	0.164	0.37	0	1
Season	Summer	If date is June or July or August then = 1, else = 0	0.245	0.43	0	1
	Winter	If date is December or January or February then = 1, else = 0	0.261	0.439	0	1
Day of Week	Friday	If day of week is Friday then = 1, else = 0	0.18	0.384	0	1
Crash Location	Intersection_ 4ty	If crash occurred on four-way intersection, T-intersection and Y-intersection then = 1, else = 0.	0.422	0.494	0	1
	Intersection_ roundabout	If crash occurred on roundabout then = 1, else =0.	0.003	0.052	0	1
	Ramp	If the crash occurred on the ramp then = 1, else = 0	0.043	0.203	0	1
	Driveway	If the crash occurred on the driveway then = 1, else =0	0.037	0.189	0	1
	Curve	If road contour is curved then = 1, else = 0	0.084	0.277	0	1
Light Condition	Ice	If road condition is ice or snow then = 1, else = 0	0.06	0.238	0	1
	Dark	If light condition is lighted roadway, roadway not lighted and unknown roadway lighting then = 1, else = 0	0.229	0.42	0	1
Speed	Speed 45	If the crash occurred on the road under posted speed 45 then = 1, else = 0	0.527	0.499	0	1
Other Crash Factors	Work zone	If the crash occurred within a work zone then = 1, else = 0	0.013	0.114	0	1
	Alcohol	If the driver was influenced by alcohol then = 1, else = 0	0.049	0.216	0	1
	Drug	If the driver was influenced by drugs then = 1, else = 0	0.011	0.102	0	1
	Pedestrian	If pedestrian was involved in the crash then = 1, else = 0	0.004	0.064	0	1

**Table 3:** Descriptive statistics of the selected TAZ-level variables.

Variable	Description	Mean	Std Dev	Minimum	Maximum
Urban	If the TAZ is urban = 1, if not = 0	0.85	0.357	0	1
Mile_	Distance to center of Columbus (mile)	9.978	8.25	0.073	44.466
Columbus					
Popdensity	Population/TAZ area (acre)	5.181	5.788	0	47.043
Hhinc10	Household income 2010	52,079.21	25,292.19	8785	161,377
Empretsrv1	Retail goods employment 2010	113.915	196.609	0	2124
WHITE_P	% of Whites in the TAZ	0.717	0.269	0	1
Punder14	% of population under 14	0.184	0.079	0	0.442
P5064	% of population between 50 and 64	0.177	0.07	0	1
POver65	% of population over 65	0.116	0.1	0	1
Agriculture	% of agricultural land use	0.137	0.275	0	1
Residential	% of residential land use	0.346	0.271	0	0.962
Built_age	Built age of construction in the TAZ where the crash occurred	44.307	23.371	5	509.25
HistoricD	If the crash occurred in historic districts = 1, if not = 0	0.021	0.143	0	1
Shop_acre	Area of Shopping Centers	3.432	9.085	0	76.859

**Table 4:** Logit estimation results.

Variable	DF	Estimate	Standard Error	Wald Chi-Square	Pr > ChiSq
Intercept	1	1.0766	0.094	130.3	<0.0001
**Crash-Related Factors**					
AG	1	−0.0134	0.006	5.26	0.0219
AG^2^	1	0.000344	0.0001	6.64	0.01
AG^3^	1	−0.00000285	0.00000092	9.46	0.0021
MD	1	0.1269	0.012	116.73	<0.0001
Compact	1	−0.3152	0.031	102.1	<0.0001
MidSize	1	−0.331	0.03	124.17	<0.0001
FullSize	1	−0.3033	0.033	84.89	<0.0001
Van	1	−0.3295	0.034	95.49	<0.0001
SUV	1	−0.39	0.031	157.35	<0.0001
Pickup	1	−0.2699	0.033	68.94	<0.0001
Motorcycle	1	−2.9743	0.093	1032.12	<0.0001
Bus	1	0.3928	0.092	18.15	<0.0001
PeakTime1	1	0.0619	0.021	8.91	0.0028
PeakTime2	1	0.0821	0.017	22.38	<0.0001
Afternoon	1	0.033	0.015	4.6	0.032
Summer	1	−0.0612	0.014	20.06	<0.0001
Winter	1	0.153	0.014	118.38	<0.0001
Friday	1	0.0422	0.015	8.37	0.0038
Intersection_4ty	1	−0.2163	0.012	306.18	<0.0001
Intersection_roundabout	1	1.1475	0.152	56.7	<0.0001
Ramp	1	0.1664	0.029	31.92	<0.0001
Driveway	1	−0.0542	0.031	3.07	0.0799
Curve	1	−0.1925	0.021	85.77	<0.0001
Ice	1	0.2259	0.04	32.04	<0.0001
Dark	1	−0.0551	0.017	11.18	0.0008
Speed45	1	0.4173	0.013	1040.73	<0.0001
Work zone	1	0.1944	0.05	14.92	0.0001
Alcohol	1	−0.4581	0.027	288.72	<0.0001
Drug	1	−0.4678	0.053	77.93	<0.0001
Pedestrian	1	−3.3078	0.132	630.66	<0.0001
**TAZ- Related Factors**					
Urban	1	0.0936	0.022	18.55	<0.0001
Mile_Columbus	1	0.00788	0.001	51.49	<0.0001
Popdensity	1	0.0133	0.001	79.92	<0.0001
Hhinc10	1	0.00000086	0.0000003	8.38	0.0038
Empretsrv1	1	0.000258	0.00004	47.4	<0.0001
WHITE_P	1	0.233	0.028	71.55	<0.0001
Punder14	1	−0.6121	0.086	50.64	<0.0001
P5064	1	−0.5742	0.092	38.95	<0.0001
POver65	1	−0.3184	0.06	28.07	<0.0001
Agriculture	1	−0.2358	0.036	42.54	<0.0001
Residential	1	−0.1655	0.03	30.7	<0.0001
Built_age	1	−0.00087	0.0003	9.29	0.0023
HistoricD	1	0.2065	0.045	20.93	<0.0001
Shop_acre	1	0.00145	0.001	3.71	0.0539

## Data Availability

The data that support the findings of this study are available on request from the corresponding author.
